# Effect of a cachectic factor on carbohydrate metabolism and attenuation by eicosapentaenoic acid

**DOI:** 10.1038/sj.bjc.6690490

**Published:** 1999-06

**Authors:** H J Hussey, M J Tisdale

**Affiliations:** Pharmaceutical Sciences Institute, Aston University, Birmingham, B4 7ET, UK

**Keywords:** proteolysis-inducing factor (PIF), glucose utilization, eicosapentaenoic acid (EPA)

## Abstract

The effect of a proteolysis-inducing factor (PIF), produced by cachexia-inducing tumours on glucose utilization by different tissues and the effect of pretreatment with the polyunsaturated fatty acid eicosapentaenoic acid (EPA), has been determined using the 2-deoxyglucose tracer technique. Mice receiving PIF showed a profound depression of body weight (2.3 g) over a 24-h period, which was completely abolished by pretreatment with a monoclonal antibody to PIF or by 3 days pretreatment with EPA at 500 mg kg^−1^. Animals receiving PIF exhibited a marked hypoglycaemia, which was effectively reversed by both antibody and EPA pretreatment. There was an increase in glucose utilization by brain, heart and brown fat, but a decrease by kidney, white fat, diaphragm and gastrocnemius muscle after administration of PIF. Changes in organ glucose consumption were attenuated by either monoclonal antibody, EPA, or both. There was a decrease in 2-deoxyglucose uptake by C_2_ C_12_ myoblasts in vitro, which was attenuated by EPA. This suggests a direct effect of PIF on glucose uptake by skeletal muscle. These results suggest that in addition to a direct catabolic effect on skeletal muscle PIF has a profound effect on glucose utilization during cachexia. © 1999 Cancer Research Campaign


British Journal of Cancer (1999) **80**(8), 1231–1235
© 1999 Cancer Research Campaign
Article no. bjoc.1999.0490

### Effect of a cachectic factor on carbohydrate metabolism and attenuation by eicosapentaenoic acid

**HJ Hussey and MJ Tisdale**

Pharmaceutical Sciences Institute, Aston University, Birmingham B4 7ET, UK

**Summary** The effect of a proteolysis-inducing factor (PIF), produced by cachexia-inducing tumours on glucose utilization by different tissues and the effect of pretreatment with the polyunsaturated fatty acid eicosapentaenoic acid (EPA), has been determined using the 2-deoxyglucose tracer technique. Mice receiving PIF showed a profound depression of body weight (2.3 g) over a 24-h period, which was completely abolished by pretreatment with a monoclonal antibody to PIF or by 3 days pretreatment with EPA at 500 mg $kg^{-1}$. Animals receiving PIF exhibited a marked hypoglycaemia, which was effectively reversed by both antibody and EPA pretreatment. There was an increase in glucose utilization by brain, heart and brown fat, but a decrease by kidney, white fat, diaphragm and gastrocnemius muscle after administration of PIF. Changes in organ glucose consumption were attenuated by either monoclonal antibody, EPA, or both. There was a decrease in 2-deoxyglucose uptake by $C_2C_{12}$ myoblasts in vitro, which was attenuated by EPA. This suggests a direct effect of PIF on glucose uptake by skeletal muscle. These results suggest that in addition to a direct catabolic effect on skeletal muscle PIF has a profound effect on glucose utilization during cachexia.

**Keywords:** proteolysis-inducing factor (PIF); glucose utilization; eicosapentaenoic acid (EPA)

Patients with cancer frequently experience a debilitating and terminal weight-losing syndrome known as cancer cachexia with depletion of both adipose tissue and skeletal muscle mass. Various mediators have been postulated to be responsible for this condition, including cytokines such as tumour necrosis factor alpha (TNF-α), interleukin-6 (IL-6) and interferon gamma (IFN-γ), together with tumour-produced catabolic factors acting on adipose tissue and skeletal muscle (Tisdale, 1997).

We have recently isolated and purified a sulphated glycoprotein of apparent molecular weight 24 000 from both a cachexia-inducing murine tumour (MAC16) and from the urine of patients with cancer cachexia (Todorov et al, 1996*a*; Cariuk et al, 1997). This material produces a state of cachexia when administered to non tumour-bearing mice, with specific depletion of the non-fat carcass mass (Todorov et al, 1996*a*). Unlike the cytokines the sulphated glycoprotein produced direct proteolysis in isolated skeletal muscle (Lorite et al, 1997), and for this reason it has been called proteolysis-inducing factor (PIF). The specificity of the action of PIF could be demonstrated by the neutralization of the catabolic action on muscle by a monoclonal antibody originating from the splenocytes of mice transplanted with the MAC16 tumour (Todorov at al, 1996*b*). In addition, protein degradation initiated in vitro by PIF was aboloshed by using skeletal muscle from mice treated with the polyunsaturated fatty acid, eicosapentaenoic acid (EPA) (Lorite et al, 1997), which has been shown to attenuate the development of cachexia, not only in the MAC16 model (Tisdale and Beck, 1991), but also in patients with unresectable pancreatic cancer (Wigmore et al, 1996).

In vivo studies with both murine (Todorov et al, 1996*a*) and human (Cariuk et al, 1997) PIF demonstrated a marked depression in blood glucose levels, in addition to the loss of lean body mass, suggesting an effect on glucose utilization. This study investigates the effect of PIF on glucose utilization by different tissues in vivo using the 2-deoxyglucose tracer technique and the effect of EPA and the monoclonal antibody on this process.

*Received 2 October 1998*
*Revised 7 January 1999*
*Accepted 27 January 1999*

*Correspondence to:* MJ Tisdale

### MATERIALS AND METHODS

### Animals

Pure strain NMRI mice were obtained from our own inbred colony and were fed a rat and mouse breeding diet (Special Diet Services, Witham, UK). Fragments of the MAC16 tumour, excised from donor animals with established weight loss, were implanted into the flanks of NMRI mice by means of a trochar (Beck and Tisdale, 1987). Tumours, which were excised from mice when they weighed between 0.1 and 0.6 g and before weight loss exceeded 25% of the total body weight, were used to prepare PIF. Solid MAC16 tumours were homogenized followed by ammonium sulphate precipitation (40% w/v), centrifuged and the supernatant was subject to affinity chromatography as previously described (Todorov et al, 1996b).

### Chemicals

2-Deoxy-D-[2,6-$^3$H]glucose ([$^3$H]2DG; sp.act. 42 mCi $mmol^{-1}$) and 2-deoxy-D-[ 1-$^{14}$C]glucose ([$^{14}$C]2DG; sp.act. 56 mCi $mmol^{-1}$) were purchased from Amersham (International, Bucks, UK). Blood glucose was determined using the o-toluidine reagent kit from Sigma Chemical Co (Dorset, UK). EPA (95%, expressed as a percentage of fatty acid methyl esters prepared) was kindly donated by Scotia Pharmaceuticals Ltd. (Stirling, UK). Hi-safe 3 scintillation fluid was from Fisons Scientific Equipment (Leicestershire, UK).

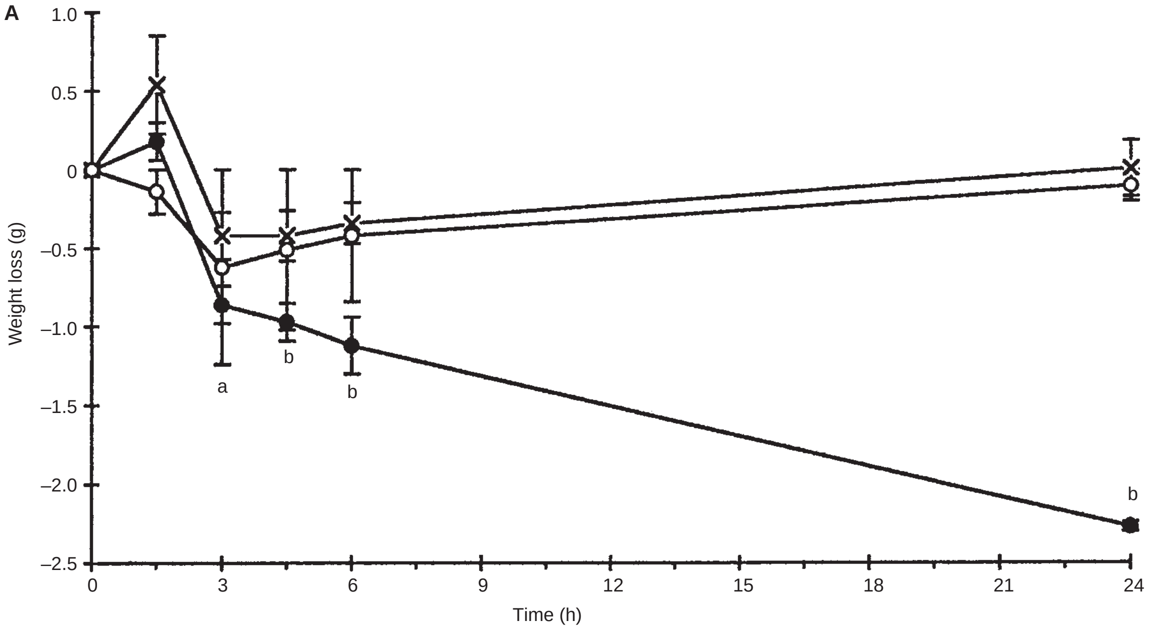

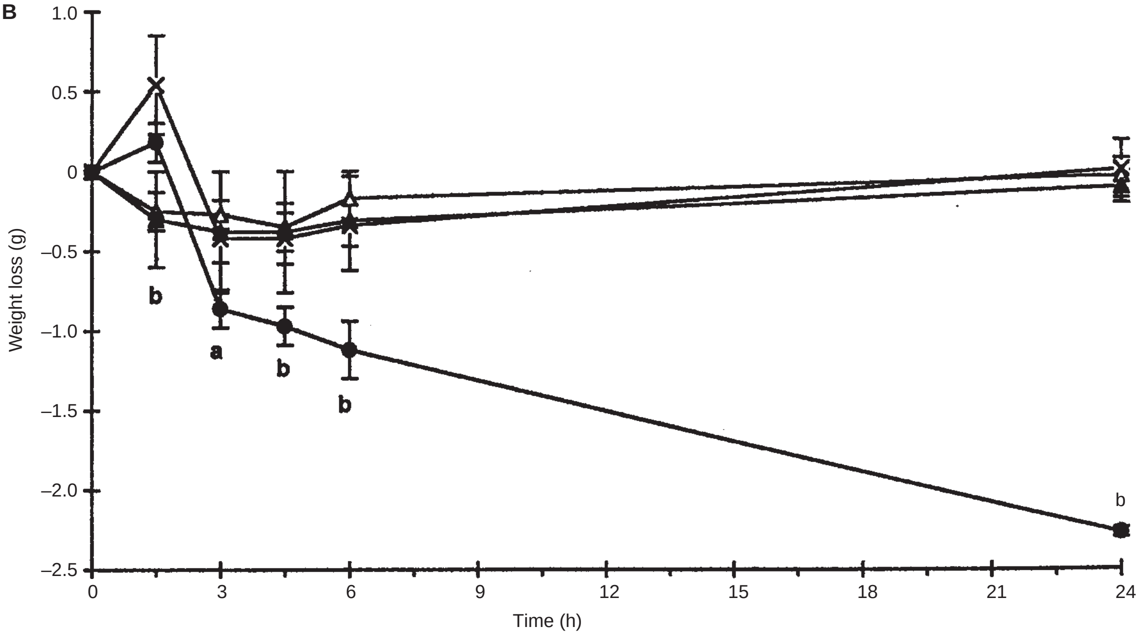

**Figure 1** Effect of PIF on body weight of female NMRI mice either administered alone (●) or after pretreatment with monoclonal antibody (○) (32 mg kg$^{-1}$) (**A**) or EPA (Δ) (500 mg kg$^{-1}$) (**B**). Control mice (×) in both groups received PBS, while in **B** an additional control group received EPA (▲) (500 mg kg$^{-1}$). PIF was administered at time 0, 1.5, 3.0, 4.5 and 6.0 h as described in Methods. The starting weight of the mice was 24.85 ± 0.38 g. Weights are expressed as mean ± s.e.m. for five animals per group. Differences from the control group were determined using two-way ANOVA followed by Tuckey's test and are indicated as 'a' $P < 0.01$ and 'b' $P < 0.005$

### Glucose utilization in vivo

The extent of glucose utilization by different tissues was determined in female NMRI mice after administration of PIF using the 2-deoxyglucose tracer technique (Meszaros et al, 1987), except that animals were not starved. One group of mice received EPA, per orally (p.o.) at 500 mg$^{-1}$ kg for 3 days prior to PIF, whilst another received monoclonal antibody, prepared as previously described (Todorov et al, 1996*b*), by intraperitoneally (i.p.) administration of 0.8 mg protein in 100 μl phosphate-buffered saline (PBS) 24 h before the first injection of PIF. Animals were administered PIF (7.5 μg protein administered by injection into the tail vein) at 0, 1.5, 3.0, 4.5 and 6.0 h whilst controls were administered PBS by the same route. At 24 h after the first injection of PIF all animals were injected i.v. with 30 μCi [$^3$H]2DG, and to determine

 © 1999 Cancer Research Campaign

**Table 1** Effect of PIF on blood glucose levels in female NMRI mice[a]

| Group | Glucose nmol $ml^{-1}$ (mean ± s.e.m.) |
|---|---|
| PBS | 9.51 ± 0.45 |
| EPA | 11.54 ± 0.79[b] |
| PIF | 6.51 ± 0.57 |
| PIF + Ab | 8.57 ± 0.30[c] |
| PIF + EPA | 10.35 ± 0.38[c] |

[a]Blood glucose values were determined 24 h after the first injection. The experimental procedure is described in Methods and the legend to Figure 1. Statistical analysis was by Student's *t*-test with Bonferroni correction. [b]$P < 0.005$ from PBS control. [c]$P < 0.005$ from PIF. PBS, phosphate-buffered saline; EPA, eicosapentaenoic acid; PIF, proteolysis-inducing facter; Ab, antibody.

**Table 2** Tissue glucose metabolic rate (Rg)

| Tissue | Control | PIF | PIF + Ab | EPA | PIF + EPA |
|---|---|---|---|---|---|
| Brain | 70 ± 10[c] | 230 ± 10[f] | 80 ± 10[c] | 200 ± 60[b,f] | 80 ± 10[c] |
| Heart | 84 ± 9[c] | 170 ± 8[f] | 137 ± 11[c,f] | 138 ± 18[c,f] | 60 ± 5[c,d] |
| Liver | 48 ± 2 | 47 ± 3 | 14 ± 3[c,e] | 62 ± 6 | 25 ± 4[a,e] |
| Kidney | 52 ± 3[a] | 26 ± 4[d] | 24 ± 4[c,e] | 92 ± 13[c,f] | 55 ± 6[b] |
| Spleen | 61 ± 3 | 43 ± 5 | 20 ± 1[a,f] | 66 ± 4[a] | 47 ± 3 |
| White fat | 30 ± 2 | 11 ± 1 | 31 ± 10 | 33 ± 2[a] | 47 ± 6[c] |
| Brown fat | 75 ± 3[c] | 111 ± 7[f] | 50 ± 3[c,d] | 98 ± 16[d] | 100 ± 12[d] |
| Diaphram | 174 ± 25[c] | 53 ± 5[f] | 61 ± 3[f] | 168 ± 16[c] | 201 ± 16[c,d] |
| Gastrocnemius | 89 ± 10[c] | 17 ± 1[f] | 41 ± 2[a,f] | 99 ± 11[c] | 90 ± 10[c] |

Rg values are expressed as nmol glucose $g^{-1}$ $min^{-1}$ and are given as mean ± s.e.m. Values were determined 24 h after the first injection of PIF. Statistical analysis was performed using two-way ANOVA followed by Tuckey's test. Differences are expressed as [a]$P < 0.05$, [b]$P < 0.01$ and [c]$P < 0.005$ from the PIF-treated group and [e]$P < 0.01$ and [f]$P < 0.005$ from the PBS control group. PIF, proteolysis-inducing factor, Ab, antibody; EPA, eicosapentaenoic acid.

the retention of 2-deoxyglucose 6-phosphate (2DGP) by different tissues a second i.v. injection of 3 μCi [$^{14}$C]2DG was administered 35 min after the injection of [$^{3}$H]2DG. Blood was removed from animals under anaesthesia by cardiac puncture at 60 min and the glucose concentration in whole blood was determined using the o-toluidine reagent kit. The concentration of radioactivity in the blood was determined on deproteinized neutralized samples with a dual $^{3}$H/$^{14}$C analyser. The accumulation of phosphorylated metabolites of 2DG was measured in selected tissues. Each tissue was homogenised in 0.4–0.8 ml per 100 mg tissue of ice-cold 0.5 N perchloric acid, followed by centrifugation. The supernatant was neutralized to pH 7 with 10% w/v potassium hydroxide and the radioactivity was determined after the removal of the insoluble potassium perchlorate. This gave a measure of the total 2DG radioactivity in the tissue. 2DGP was removed from the neutral extract by precipitation with zinc sulphate/barium hydroxide and the difference between the total radioactivity of the neutral extract and that after removal of 2DGP represented the 2DGP content of the tissue.

Glucose utilization was calculated from the equation (Meszaros et al, 1987):

$$Rg = Cm^*(T)/LC \int_o^T \frac{Cp^*}{Cp} \cdot dt$$

where Rg is the tissue glucose metabolic rate (nmol $min^{-1}$ $g^{-1}$), Cm*(T) is the concentration of phosphorylated metabolites of 2DG in the tissue (dpm $g^{-1}$) at t = 60 min, Cp is the blood glucose (nmol $ml^{-1}$), Cp* is the concentration of [$^{3}$H]2DG in the blood (dpm $ml^{-1}$) and LC (lumped constant) is a dimensionless correction factor for discrimination against 2DG in glucose metabolic pathways. This was determined by the method of Ferre et al (1985) and found to be 0.46 in NMRI mice (Mahony and Tisdale, 1990).

### Effect of PIF on glucose utilization in vitro

Experiments were performed on the $C_2C_{12}$ mouse myoblast cell line in the subconfluent state. Cells were seeded in 60 × 15 mm petri dishes at 4 × $10^4$ cells $ml^{-1}$ in 2 ml Dulbecco's modified Eagles medium supplemented with 12% fetal calf serum, 1% non-essential amino acids and 1% pencillin–streptomycin and left for 24 h. Some cultures received 50 μM EPA (dissolved g for g in bovine serum albumin then mole for mole in sodium bicarbonate and sonicated to produce micelles). After 2 h PIF was added in phosphate-buffered saline (PBS) and the cultures left for 24 h. The medium was aspirated off and the cells were washed once with Krebs–Ringer bicarbonate buffer, pH 7.2, and incubated in 1 ml of the Krebs buffer for 30 min at 37°C together with 10 μl [$^{3}$H]2DG (sp. act, 2 mCi $mmol^{-1}$). The cells were washed three times with ice-cold PBS and incubated on ice for at least 1 h with 1 M sodium hydroxide (1 ml per dish). The radioactivity was determined in Hi-safe 3 scintillation fluid using a 2000 CA Tri-Carb liquid scintillation analyser.

### RESULTS

The effect of PIF administered either alone or after pretreatment with the monoclonal antibody (Figure 1A) or EPA (Figure 1B) on the body weight of mice was determined over a 24 h period. There was a rapid decrease in body weight of mice receiving PIF, which first became significant within 3 h after the first injection and reached 2.3 g by 24 h. Previous studies (Todorov et al, 1996*a*; Lorite et al, 1997) have shown that this loss of body weight is due to loss of the non-fat carcass mass, predominantly skeletal muscle. Pretreatment of mice with either the monoclonal antibody (Figure 1A) or EPA (Figure 1B) completely abolished the cachectic effect of PIF and produced a weight loss profile indistinguishable from PBS- or EPA-treated control groups. Both the antibody and EPA were equieffective in reversing the effect of PIF on body weight loss.

In order to investigate glucose dynamics in organs of mice treated with PIF, glucose utilization was investigated by the 2DG tracer method. Animals treated with PIF showed a marked hypoglycaemia within 24 h of the first injection (Table 1), which was effectively reversed with both antibody and EPA pretreatment. The tissue glucose metabolic rate (Rg) of saline control and PIF-treated animals, with or without antibody and EPA pretreatment, together with EPA controls is shown in Table 2. The Rg values were lower than those we have previously reported (Mahony and Tisdale, 1990; Mulligan and Tisdale, 1991) since the animals were not starved overnight, and throughout the experiment because of the profound weight loss induced by PIF. There were no differences in food intake (2.5, 2.7 and 2.8 g per mouse for control, PIF and PIF + EPA groups respectively) between the groups as previously reported (Todorov et al, 1996*a*; Lorite et al, 1997). There were major changes in the Rg values in a number of organs after administration of PIF, which were attenuated by either the antibody or EPA or both (Table 2).

*© 1999 Cancer Research Campaign*

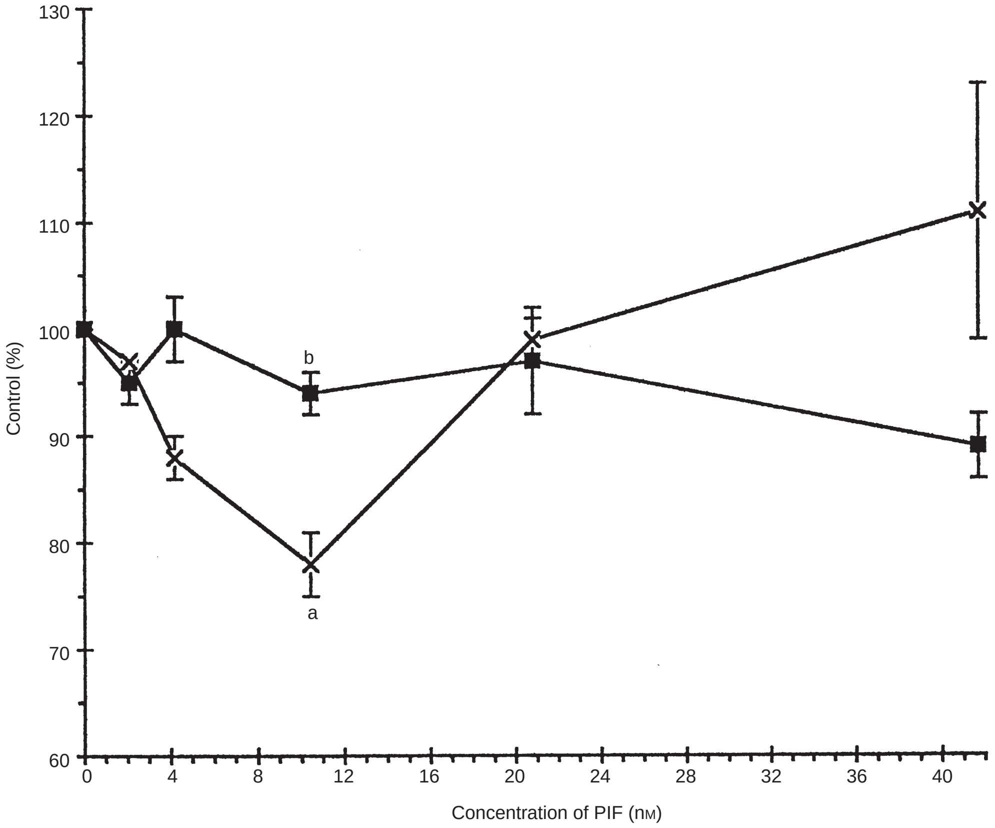

**Figure 2** Effect of PIF on uptake of 2-deoxyglucose into $C_2C_{12}$ myoblasts in vitro. Cells were incubated with PIF with (■) or without (×) pretreatment with 50 µM EPA. Results are shown as mean ± s.e.m. where $n = 9$. Differences from controls are indicated as 'a' $P < 0.05$, while differences from the PIF alone treated group are shown as 'b' $P < 0.05$

### Brain

There was a threefold increase in the Rg value for glucose in animals treated with PIF, which was reduced to control values in mice pretreated with both EPA and antibody, despite the elevation in Rg value for mice treated with EPA alone.

### Heart

There was a twofold elevation in the Rg value for glucose in mice treated with PIF, which was reduced to below control value by EPA, despite a significant elevation in Rg value producted by EPA alone. There was a significant reduction in Rg value produced by the antibody, although this still remained elevated compared with saline treated controls.

### Liver

Although PIF alone produced no alteration in the Rg value for glucose, the combination of PIF with either the antibody or EPA caused a significant reduction in Rg value to below that of control animals.

### Kidney

There was a 50% reduction in the Rg value for glucose in animals treated with PIF, which was normalized to the control value in mice pretrated with EPA, but not the antibody. Again, EPA alone produced a significant increase in the Rg value.

### Spleen

There was a small decrease in the Rg value in PIF-treated animals, which did not reach significance. The combination of PIF and the antibody produced a significant decrease in Rg value to below that of the control.

### White fat

There was a decrease in Rg value in animals treated with PIF, although this was not significantly different from the control. Animals pretreated with EPA before receiving PIF had a Rg value significantly higher than those receiving PIF alone.

### Brown fat

There was a significant elevation in the Rg value in animals receiving PIF, which was reduced to below the control level by the antibody, but not by EPA.

### Diaphragm

There was a large reduction in the Rg value produced by PIF, which was normalized by EPA, but not by the antibody.

*© 1999 Cancer Research Campaign*

### Gastrocnemius muscle

There was an 80% reduction in the Rg value in animals treated with PIF, which was attenuated by both the antibody and EPA.

PIF produced a decrease in uptake of 2-deoxyglucose in $C_2C_{12}$ myoblasts in vitro, a model of skeletal muscle (Figure 2). This effect was maximal at 10 nM PIF and was not seen in cells pretreated with EPA. This suggests that PIF has a direct inhibitory effect on glucose consumption by diaphragm and gastrocnemius muscle in vivo.

### DISCUSSION

There have been very few reports on glucose oxidative metabolism in cachectic cancer patients. Waterhouse and Kemperman (1971) observed that in the post-adsoptive state both cachectic cancer patients and controls showed similar oxidative metabolism from glucose and fatty acids. However, after a glucose load, oxidative metabolism from glucose in the control subjects increased by 62% and that from fatty acids fell by 70%, but there was little change in the oxidative metabolism pattern in the cachectic patients. This suggests that weight-losing cancer patients may maintain a fasting oxidative metabolism profile, even in the presence of a glucose load. There was also a slower glucose disappearance from the blood after a glucose load in the cachectic cancer patients. This may be indicative of reduced uptake of glucose by peripheral tissues as also observed by other workers (Schein et al, 1979), suggesting a degree of glucose intolerance in patients with cancer cachexia. Reduced utilization of glucose by peripheral tissues was also suggested from the observation of a decrease in activity of glycolytic and glucose oxidative enzymes in biopsies from rectus abdominal muscle of patients with heterogenous malignancies (Lundholm et al, 1976).

The results of the present investigation suggest profound changes in glucose homeostasis after administration of PIF, with hypoglycaemia and specific tissue alterations in glucose consumption. Hypoglycaemia has previously been reported with both human (Nolop et al, 1987) and experimental tumours such as the MAC16 adenocarcinoma (McDevitt and Tisdale, 1992) and appears to be unrelated to food intake, glucose consumption by the tumour or insulin-like growth factors (either IFG-I or IGF-II) produced by tumours. Insulin levels have been found to be low in cases of tumour-associated hypoglycaemia (Bibby et al, 1987) and the production of an insulin-like factor by the tumour was suggested as the cause of the enhanced peripheral glucose uptake. The present results suggest that this mediator is PIF, although glucose consumption was found to be significantly increased only in brain, brown fat and heart, while in skeletal muscles, such as gastrocnemius and diaphragm, glucose consumption was found to be significantly decreased. This is consistent with the observation that PIF produces a significant decrease in the weight of the soleus and gastrocnemius muscles, while having no effect on the weight of the heart (Lorite et al, 1998).

Although the action of PIF on body weight loss appears to be mediated through skeletal muscle protein degradation (Todorov et al, 1996*b*), an increased glucose consumption by brown fat is indicative of an increased energy expenditure, which may play a contributory role in the overall development of weight loss induced by PIF. EPA also increased glucose consumption in brown fat, which would correlate with the report of a marked stimulation of brown adipose tissue (BAT) thermogenic activity by n-3 polyunsaturated fatty acids (Oudart et al, 1997).

The effect of PIF on both body weight loss and glucose utilization was attenuated by treatment both with a monoclonal antibody to PIF and EPA showing the specificity of the process. EPA has been shown to inhibit the biological activity of both a lipid mobilizing factor (Tisdale and Beck, 1991) and PIF (Lorite et al, 1997) in bioassays, as well as attenuating the development of cachexia in the MAC16 model. The ability of EPA to prevent the development of weight loss initiated by PIF confirms a direct antagonistic effect rather than by an effect on the biosynthesis of PIF. PIF appears to have a direct inhibitory effect on glucose consumption by skeletal muscle, since uptake of 2-deoxyglucose by $C_2C_{12}$ myoblasts in vitro was also inhibited by PIF. This suggests that pertubations in glucose homeostatis in animals receiving PIF is not due to release of cytokines.

### REFERENCES

Beck SA and Tisdale MJ (1987) Production of lipolytic and proteolytic factors by a murine tumor-producing cachexia in the host. *Cancer Res* **47**: 5919–5923

Bibby MC, Double JA, Ali SA, Fearon KCH, Brennan RA and Tisdale MJ (1987) Characterization of a transplantable adenocarcinoma of the mouse colon producing cachexia in recipient animals. *J Natl Cancer Inst* **78**: 539–546

Cariuk P, Lorite MJ, Todorov PT, Field WN, Wigmore SJ and Tisdale MJ (1987) Induction of cachexia in mice by a product isolated from the urine of cachectic cancer patients. *Br J Cancer* **76**: 606–613

Ferre P, Leturgue A, Bumol A-P, Penicaud L and Girad J (1985) A method to quantify glucose utilization in vivo in skeletal muscle and white adipose tissue of the anaesthetized rat. *Biochem J* **228**: 103–110

Lorite MJ, Cariuk P and Tisdale MJ (1997) Induction of muscle protein degradation by a tumour factor. *Br J Cancer* **76**: 1035–1040

Lorite MJ, Thompson MG, Drake JL, Carling G and Tisdale MJ (1998) Mechanism of muscle protein degradation induced by a cancer cachectic factor. *Br J Cancer* **78**: 850–856

Lundholm K, Bylund AC, Holm J and Schersten T (1976) Skeletal muscle metabolism in patients with malignant tumour. *Eur J Cancer* **12**: 465–473

McDevitt TM and Tisdale MJ (1992) Tumour-associated hypoglycaemia in a murine cachexia model. *Br J Cancer* **66**: 815–820

Mahony SM and Tisdale MJ (1990) Metabolic effects of tumour necrosis factor alpha in NMRI mice. *Br J Cancer* **61**: 514–519

Meszaros K, Bagby G, Lang C and Spitzer JJ (1987) Increased uptake and phosphorylation of 2-deoxyglucose by skeletal muscles in endotoxin-treated rats. *Am J Physiol* **253**: 33–39

Mulligan HD and Tisdale MJ (1991) Metabolic substrate utilization by tumour and host tissues in cancer cachexia. *Biochem J* **277**: 321–326

Nolop KB, Rhodes CG, Brudin LH, Beaney RP, Kravsz T, Jones T and Hughes JM (1987) Glucose utilisation in vivo by human pulmonary neoplasms. *Cancer* **60**: 2682–2689

Oudart H, Groscolas R, Calgari C, Nibbelink M, Leray C, Maho Y Le and Malan A (1997) Brown fat thermogenesis in rats fed high-fat diets enriched with n-3 polyunsaturated fatty acids. *Int J Obesity* **21**: 955–962

Schein PS, Kiener D, Haller D, Beecher M and Hamosh M (1979) Cachexia of malignancy. Potential role of insulin in nutrional management. *Cancer* **43**: 2070–2076

Tisdale MJ (1997) Biology of cachexia. *J Natl Cancer Inst* **89**: 1763–1773

Tisdale MJ and Beck SA (1991) Inhibition of tumour-induced lipolysis *in vitro* and cachexia and tumour growth in vivo by eicosapentaenoic acid. *Biochem Pharm* **41**: 103–107

Todorov PT, Cariuk P, McDevitt TM, Coles B, Fearon K and Tisdale MJ (1996*a*) Characterization of a cancer cachectic factor. *Nature* **379**: 739–742

Todorov PT, McDevitt TM, Cariuk P, Coles B, Deacon M and Tisdale MJ (1996*b*) Induction of muscle protein degradation and weight loss by a tumor product. *Cancer Res* **56**: 1256–1261

Wigmore SJ, Ross JA, Falconer JS, Plester CE, Tisdale MJ, Carter DC and Fearon KCH (1996) The effect of polyunsaturated fatty acids on the progress of cachexia in patients with pancreatic cancer. Nutrition **12**: S27–S30

© 1999 Cancer Research Campaign
